# Supplementary material for: Meta-Analysis of Survival and Development of a Prognostic Nomogram for Malignant Pleural Mesothelioma Treated with Systemic Chemotherapy
Source: Cancers (Basel). 2021 May 2;13(9):2186. doi: 10.3390/cancers13092186 (PMC8124134; doi:10.3390/cancers13092186)
Supplement: Supplementary file 1 [file cancers-13-02186-s001.zip › cancers-1187334-supplementary.pdf]

cancers MDPI

Supplemental Materials

Meta-Analysis of Survival and Development of a Prognostic Nomogram for Malignant Pleural Mesothelioma Treated with Systemic Chemotherapy

Rupesh Kotecha, Raees Tonse, Muni Rubens, Haley Appel, Federico Albrecht, Paul Kaywin, Evan W. Alley, Martin C. Tom and Minesh P. Mehta

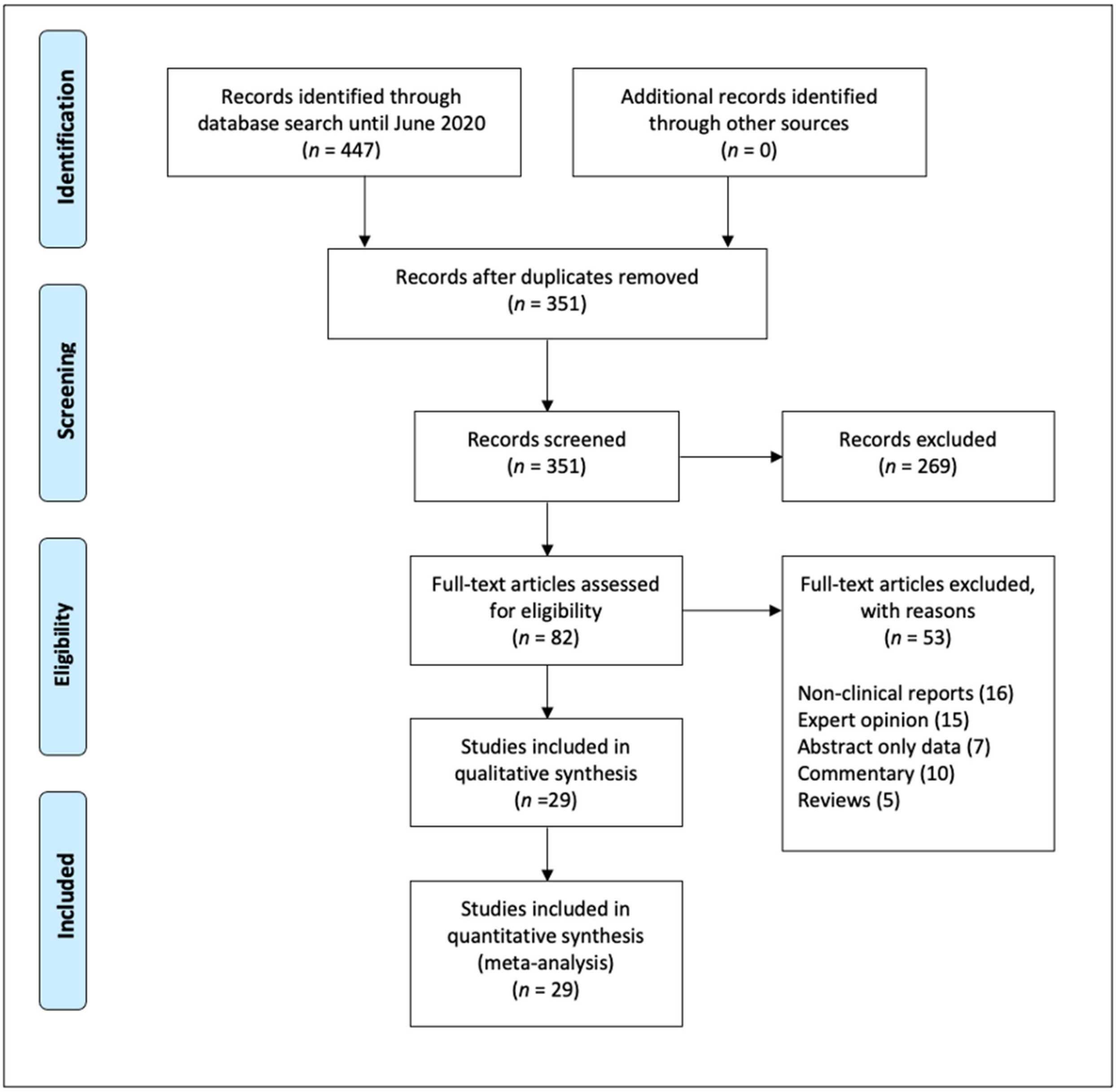

Figure S1. PRISMA flow diagram showing the selection of studies for the systematic review of malignant pleural mesothelioma patients treated in the first-line setting with combination chemotherapy regimens.

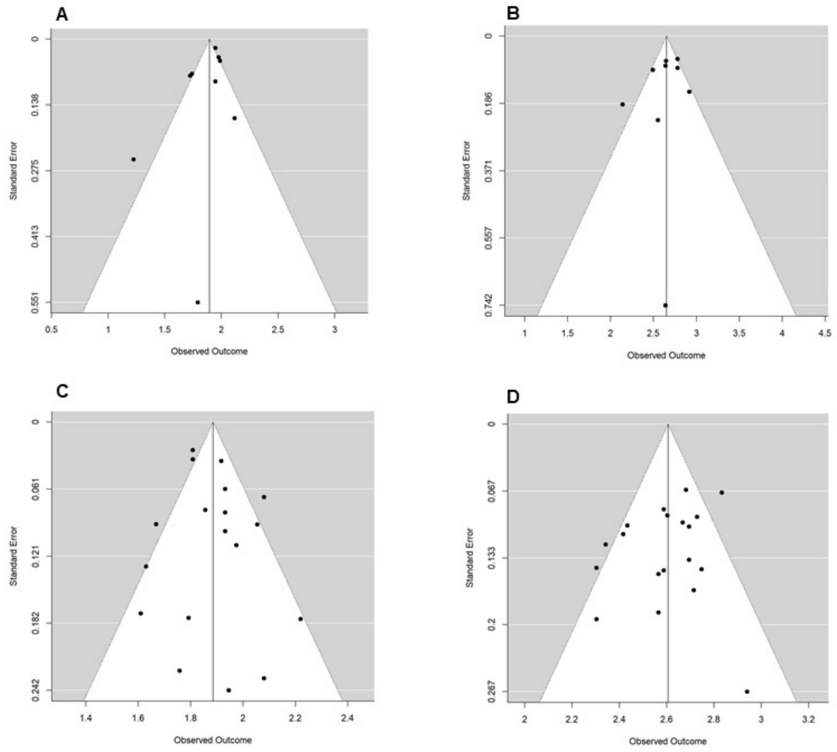

Figure S2. Funnel plots for (**A**) progression-free survival with platinum/pemetrexed, (**B**) overall survival with platinum/pemetrexed, (**C**) progression-free survival with other experimental therapies, and (**D**) overall survival with other experimental therapies to assess the potential for publication bias. The measure of effect size and study precision are shown on *x*- and *y*-axis.
